# Neural Oscillatory Correlates for Conditioning and Extinction of Fear

**DOI:** 10.3390/biomedicines6020049

**Published:** 2018-05-01

**Authors:** Carlos Trenado, Nicole Pedroarena-Leal, Laura Cif, Michael Nitsche, Diane Ruge

**Affiliations:** 1 Department of Psychology and Neurosciences, Translational Neuromodulation Unit, Leibniz Centre for Working Environment and Human Factors, Technical University Dortmund, 44227 Dortmund, Germany; nicole.pedroarena.13@ucl.ac.uk (N.P.-L.); nitsche@ifado.de (M.N.); 2 Institute of Clinical Neuroscience and Medical Psychology, Medical Faculty, Heinrich Heine University, 40225 Dusseldorf, Germany; 3 Departement de Neurochirurgie, Centre Hospitalier Universitaire Montpellier, Université Montpellier, 34000 Montpellier, France; a-cif@chu-montpellier.fr

**Keywords:** oscillations, extinction learning, fear conditioning, fear extinction, translational

## Abstract

The extinction of conditioned-fear represents a hallmark of current exposure therapies as it has been found to be impaired in people suffering from post-traumatic stress disorder (PTSD) and anxiety. A large body of knowledge focusing on psychophysiological animal and human studies suggests the involvement of key brain structures that interact via neural oscillations during the acquisition and extinction of fear. Consequently, neural oscillatory correlates of such mechanisms appear relevant regarding the development of novel therapeutic approaches to counterbalance abnormal activity in fear-related brain circuits, which, in turn, could alleviate fear and anxiety symptoms. Here, we provide an account of state-of-the-art neural oscillatory correlates for the conditioning and extinction of fear, and also deal with recent translational efforts aimed at fear extinction by neural oscillatory modulation.

## 1. Introduction

Pavlovian conditioning involves a conditioned stimulus (CS) that is paired with an unconditioned stimulus (US). The conditioned stimulus is generally neutral while the unconditioned stimulus has some affective valence. Upon repeated presentation of a CS-US pairing, a subject exhibits a conditioned response (CR) when the CS is presented alone. With regard to the US, a subject exhibits an unconditioned response (UR) [1]. Within the context of fear conditioning studies, an aversive stimulus (such as an electric shock or loud sound) is paired with a neutral context stimulus, resulting in the expression of a fear response in the presence of the context or stimulus alone [2].

**Figure 1:**
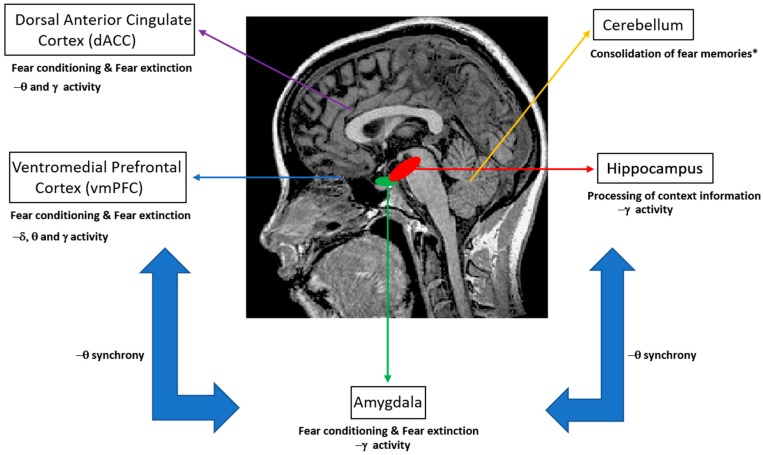
Human brain regions that have been investigated in studies of fear conditioning and fear extinction. Here, we emphasize the relevant neural oscillatory correlates (δ: delta oscillations; θ: theta oscillations; γ: gamma oscillations) of such mechanisms, as suggested by animal and human studies. * Note that the role of the cerebellum in conditioning and extinction of fear is a matter of current research. Nevertheless, functional aspects such as the consolidation of fear memories have been suggested by previous studies [11,12].

Extinction learning following Pavlovian conditioning is characterized by a gradual decrease in response to a CS that is not reinforced in subsequent presentations. In particular, fear extinction occurs when a previously fear-conditioned subject is exposed to a fear-eliciting stimulus (CS) in the absence of an aversive stimulus (US) [3]. Previous studies of extinction in nonhuman animals and humans have focused on the amygdala, the ventral medial prefrontal cortex (vmPFC), the dorsal anterior cingulate cortex (dACC), the hippocampus and the cerebellum (Figure 1) [4,5,6,7,8]. In particular, it has been shown that the amygdala is involved in the acquisition of conditioned fear as well as fear extinction [9], while animal reports emphasize the role of more specific reward circuits (the basolateral amygdala (BLA)–nucleus accumbens (NAc) circuit) that can regulate the persistence of fear extinction [10]. Interestingly, previous studies have suggested the involvement of the cerebellum in memory consolidation and neural circuits involved in fear memories [11,12].

Neuronal oscillations are considered a mechanism that synchronizes neural activity at different scales across brain regions and enhances information transfer [13,14]. As emphasized by previous accounts, the rich connectivity between neurons is a key aspect of the brain that gives rise to neural activity represented by neural oscillations, e.g., variations in time of electrical signals from neurons that can be differentiated according to their frequency: delta (0.5–4 Hz) (usually associated with deep stages of sleep), theta (4–8 Hz) (emphasized during drowsiness, arousal and meditation), alpha (8–12 Hz) (representing the default mode of the brain during states of rest and alertness), beta (12–25 Hz) (often related to active thinking and concentration, while beta oscillations recorded from the motor cortex (called mu-rhythm) are associated with muscle relaxation) and gamma (>25 Hz) (usually associated with demanding motor or cognitive functions). The synchronization of oscillations reflects temporally precise interaction of neural activities, while aberrant oscillatory synchronization has been associated with neuropsychiatric disorders, including epilepsy, schizophrenia and dementia, as well as neurodegenerative disorders, such as Parkinson’s disease [15].

In the present work, we discuss recent progress regarding neural oscillatory correlates of conditioning and extinction of fear and address translational approaches that could utilize such oscillatory findings to develop rehabilitation techniques. Specifically, we include non-invasive neuromodulation approaches such as (1) transcranial alternating current stimulation (tACS) and transcranial direct current stimulation (tDCS), which have the potential to selectively modify the brain’s cortical excitability, which has been linked to synchronization of brain networks; (2) transcutaneous vagus nerve stimulation (tVNS) which is a novel peripheral neuromodulation approach applied to the ear that has been shown to modulate the functional connectivity of brain structures such as the amygdala; (3) fMRI neurofeedback combined with perceptual learning, which relies on unconscious behavioral training aimed at modulating the neural activity of the vmPFC. As for invasive approaches, we deal with deep brain stimulation (DBS), which requires neurosurgical implantation of a neurostimulator device that sends electrical impulses through implanted electrodes, and thus, modulates the neural oscillatory activity of deep brain structures, such as the subthalamic nucleus (STN) in humans and the dorsomedial ventral striatum (dmVS) in animals.

We conclude that a proper understanding of fear extinction mechanisms and its neural correlates is crucial for the development of effective therapies for pathological fear disorders, such as post-traumatic stress disorder (PTSD) and anxiety, that result in abnormal fear extinction patterns.

## 2. Fear Conditioning Correlates

### 2.1. Animal Models

Previous experiments that have recorded local field potentials (LFP) during fear conditioning have shown synchrony between the medial pre-frontal cortex (mPFC), the amygdala and the hippocampus. In mice models, it has been reported that theta synchrony increases between the hippocampal CA1 and lateral amygdala during the consolidation and reconsolidation of fear memories [5,16], while such synchrony failed to increase during retrieval of conditioned fear after a long-time period (30 days post-training) [17]. Also, a reduction in LFP theta oscillations in the amygdala has been associated with aversive cue presentations and is strongly correlated with the amplitude of amygdala hemodynamic responses. Modulation of the amygdala by overexpression of 5-hydroxytryptamine transporter (5-HTT) impaired, but did not prevent, fear learning and significantly reduced amygdala hemodynamic responses to aversive cues [18]. In addition, multi-channel local field and unit recordings obtained from the infralimbic prefrontal cortex, the hippocampal CA1 and the lateral amygdala revealed a pattern of theta coherence (linear relationship between signals in theta band) and directionality (occurring at a specific phase as calculated by the Hilbert transform) within and across such regions, which correlated with behavioral responses. Specifically, units were synchronized in phase with theta oscillations in these brain regions during conditioned freezing, which characterizes states of fear memory retrieval [19].

Enhanced theta synchrony between the BLA and mPFC has been observed in animals that successfully recognized aversive conditions [20]. Specifically, it was observed that BLA firing activity was entrained to theta input from mPFC, thus suggesting that the BLA is selectively tuned to mPFC input, which was suggested as a potential mechanism for memory recall and response to fear [20]. Note that the amygdala output has also been reported to be implicated in the function of the mPFC [21].

It has also been reported, by using extracellular recordings under pharmacological and optogenetic manipulations, that behavioral expression of fear, as reflected by freezing, temporally coincides with internally generated 4-Hz oscillations in prefrontal–amygdala circuits, thus providing support for the proposal that an oscillatory mechanism mediates prefrontal amygdala coupling during fear behavior [22]. 

Interestingly, distinct alterations of prefrontal gamma oscillations during fear conditioning in relation to gender have been observed in rats. Such a finding appears relevant for understanding the neural basis of post-traumatic stress disorder, which is more prevalent in women and involves impaired extinction and mPFC dysfunction [23].

In primates, spiking synchrony has been shown to be associated with persistent aversive memories [24], and successful updating between fear and safety [25]. It was also shown that theta power in the amygdala and anterior cingulate cortex increase following exposure to aversive stimuli and before the delivery of aversive unconditioned stimuli, while a specific increase in theta coherence between these structures occurred during fear conditioning. Thus, the authors supported the role of theta oscillations in long-range communication between the amygdala and the anterior cingulate cortex during fear conditioning in primates [8].

With regard to gamma oscillations, mice studies have indicated that activity in the BLA is enhanced following fear conditioning [26]. In addition, a boost of gamma activity in the auditory cortex was shown at sites tuned by an aversive tone, which was followed by enhanced phase synchronization between single unit and LFP activity during tone presentation with a corresponding decrease in areas tuned away [27]. It was also shown that in the BLA, slow (40–70 Hz) and fast (70–120 Hz) gamma oscillations are coupled to distinct phases of theta activity and reflect synchronous high-frequency unit activity. During fear, the authors reported that theta-fast gamma coupling is enhanced, while fast gamma power is suppressed [28].

### 2.2. Humans

Numerous studies have found evidence for electroencephalographic (EEG) oscillations in the neural processing of stimuli perceived as threatening. In particular, previous studies showed increased gamma band activity to be involved in fear conditioning, which was accompanied by an increase in gamma-band coherence between occipital and parietal EEG electrodes [29]. Source localization studies reported theta activity of the anterior mid-cingulate cortex to be associated with fear expression [30], while an EEG controlling the arousal dimension (intensity of emotional activation) in humans was associated with an early posterior increase in theta power, followed by a later frontal increase, together with a consistent left lateralized beta desynchronization. This, emphasized the involvement of theta and beta band activity in visual threat processing [31]. Notably, cortical responses following a noxious sound paired with a visual stimulus consisting of oriented gratings were amplified and accompanied by changes in cortical connectivity between the occipital and frontal–temporal regions [32].

Recently, EEG studies have reported differential delta/theta power increases in relation to fear following conditioned and unconditioned stimuli at the prefrontal, frontal and midline channels, while a prominent alpha and beta power decrease was observed at the parietal and occipital channels early in the stimulus train. A power increase in gamma power was observed from habituation to the acquisition phase over a broad area in the frontal and occipital electrodes. The authors reported that alpha activity during fear conditioning reflects the salience and valence of conditioned/unconditioned stimuli rather than conditioning [33].

Magnetoencephalographic (MEG) studies have shown that fear conditioning leads to differences in local cortical excitability (lower alpha and beta power) between conditioned stimuli that predict the aversive unconditioned stimuli (CS+) and those that do not predict the aversive unconditioned stimuli (CS−), localized to the somatosensory cortex and insula. A graph theoretic approach also revealed the reduced connectivity of subcortical fear-related structures in the temporal and frontal areas so that the brain functional network appeared sparser during fear conditioning. The authors speculated that both reduced coupling in some regions and emerging centrality of others, contribute to the efficient processing of fear relevant information during fear learning [34]. In addition, a large cluster of sensors over the left parietal lobe and a smaller cluster of frontal sensors showing significantly less alpha power in the threat blocks than during the safe blocks was reported [35].

By recording oscillatory activity in local field potentials (LFPs) from nine epilepsy patients with intracranial depth electrodes implanted into the amygdala and the hippocampus, high gamma (70–180 Hz) oscillatory activity from the amygdala and hippocampus was apparent during the processing of fearful faces, in contrast to neutral landscapes. On the basis of Granger causality, phase slope index and phase lag analyses, the authors reported unidirectional interaction between the amygdala and hippocampus with theta/alpha oscillations in the amygdala modulating hippocampal gamma activity. The results supported the conclusion of the amygdala influencing hippocampal dynamics during fear processing by oscillatory activity [7].

## 3. Fear Extinction Correlates

### 3.1. Animal Models

In mice, theta synchronization between the hippocampal CA1 and the lateral nuclei (LA) of BLA has been shown to decrease during fear memory extinction. Moreover, theta activity of the infralimbic prefrontal cortex has been shown to be phase-locked (synchronized in phase) to CA1-LA theta activity during the retrieval and extinction of fear memory [36,37]. Other studies have reported that theta synchronization in the CA1-LA-infralimbic mPFC (IL) network declines with extinction training and is partially rebounded upon extinction recall [38]. The directionality of theta oscillations has also been emphasized during extinction by studies reporting on prefrontal cortical spike firing, leading to hippocampal and amygdala theta oscillations, which suggests that the directional dynamics of theta-entrained activity across these areas guide changes in extinction retrieval [19].

By means of single unit and local field potential recordings in behaving mice, gamma oscillatory activity was shown to be heterogeneous during extinction learning, with variations in the strength of BLA gamma power between the early and late stages of extinction, which linearly predicted the level of post-extinction spontaneous fear recovery [26].

In male mice under anesthesia, it was shown that the prelimbic (PL) and infralimbic (IL) subdivisions of the mPFC display opposing patterns of theta power during fear extinction, which may reflect new learning. Due to their physical proximity and similar input, it was surprising that LFPs were drastically different between the two regions. Interestingly, such theta power effect was not seen in female mice as they displayed heightened freezing and persistently increased mPFC theta in both PL and IL [39].

It was also shown that following extinction learning, parvalbumin-expressing (PV) interneurons enable a competing interaction between a 6–12 Hz oscillation and a fear-associated 3–6 Hz oscillation within the BLA. The loss of this competition increases the 3–6 Hz oscillatory signature, with interaction from the BLA to the medial prefrontal cortex signaling the recurrence of fear expression, which supports the role of oscillatory activity in devising therapies for disorders such as PTSD [40].

Previous studies have also stressed the involvement of gamma oscillations in the extinction of fear. In particular, PL gamma power was increased in extinction-deficient mice compared to those that successfully extinguished fear [41]. In rats, it was reported that females show persistent prelimbic (PL) gamma activation during extinction and a failure of infralimbic (IL) gamma activation during extinction recall, which does not hold for male mice. Thus, the authors emphasized that the alteration of prefrontal gamma oscillations in relation to the extinction of fear is likely to differ between genders [23].

### 3.2. Humans

Previous neuroimaging studies in humans support the involvement of the anterior mid cingulate cortex (AMC) and the ventro medial prefrontal cortex (vmPFC) in the extinction of fear. In particular, it was found that extinguished stimuli evoke a higher magnitude of vmPFC-localized gamma power in comparison to non-extinguished stimuli. In addition, subjects who failed to show a suppressed skin conductance response (i.e., reduction of fear) to the extinguished versus non-extinguished fear conditioned stimuli, also failed to show the observed alterations in vmPFC gamma power. On the basis of such results, the authors suggested that successful fear extinction recall relates to changes in vmPFC gamma power [30].

Recently, some authors compared CS presented during extinction ((CS+E, CS−E) stimuli presented without US, here CS+ and CS− refer to stimuli paired and not paired with US during the acquisition phase, respectively) and stimuli not presented during extinction ((CS+N, CS−N) stimuli whose learned responses remained intact) so as to identify effects of extinction vs. fear recall by simultaneous EEG-fMRI. These authors reported that differential (CS+ vs. CS−) electrodermal, frontomedial theta (EEG) and amygdala responses (fMRI) were reduced for extinguished versus non-extinguished stimuli. Statistical analysis revealed that the effects on theta power covaried with the effects on amygdala activation. Fear and extinction recall, as indicated by theta, explained 60% of the variance in the analogous effect in the right amygdala [42].

## 4. Summary of Oscillatory Neural Correlates

Focusing on neural correlates of fear conditioning, animal studies suggest the predominance of theta synchrony between the mPFC, hippocampal CA1 and BLA regions as well as gamma–theta coupling accompanied with gamma power suppression in the BLA, as reflected in LFPs and multiunit activity recordings. For humans, invasive LFP recordings on epilepsy patients have shown that theta/alpha activity of the amygdala modulates hippocampal gamma activity, while the predominance of gamma activity in these brain structures has been emphasized (Figure 1). Non-invasive M/EEG studies in humans have stressed the presence of delta, theta, alpha, beta and gamma oscillations at different scalp regions, namely, increased gamma oscillations at the occipital and prefrontal regions, increased theta oscillations at the posterior and latero-frontal regions, and decreased alpha and beta oscillations at the parietal and occipital regions, with the presence of such oscillations in the somatosensory cortex and insula. Importantly, the modulation of alpha activity has been attributed to the salience and valence of stimuli, rather than fear conditioning. Taken together, animal and human studies indicate that theta and gamma oscillations may provide a mechanism for the fine-tuned organization of neural pathways that are engaged in memory formation and recall of fear, by emphasizing the interaction between the mPFC, hippocampus and amygdala through theta synchrony (Figure 1).

With respect to neural correlates of fear extinction, animal studies have emphasized decreased theta synchrony between the hippocampal CA1, BLA and mPFC regions. In addition, an important role of the mPFC is indicated by the occurrence of differential theta and gamma oscillatory patterns for the PL and IL in relation to fear extinction and gender differences. Notably, the BLA has been suggested as a prospective target for the modulation of oscillatory activity with implications in the extinction of abnormal fear expression. For humans, changes in gamma power for the vmPFC and cingulate cortex as well as reductions in the theta power of the cortical frontomedial region and amygdala activation have been emphasized as markers of fear extinction. Taken together, previous findings emphasize the importance of modulating theta oscillations between the hippocampus, amygdala and mPFC regions, together with the modulation of gamma oscillations in the vmPFC as a potential neuromodulation intervention for the treatment of anxiety and fear disorders.

With regard to the functionality of the mentioned brain oscillations, it is important to clarify that neural correlates refer to observed relationships between oscillatory activity, on different scales (M/EEG, LFP, multi-unit, fMRI), and behavior (extinction and acquisition of fear), although the crucial aspect of causality is not addressed. Nevertheless, one important mission of translational approaches is to establish causality relationships between neural oscillatory activity and the acquisition/extinction of fear which could, in turn, facilitate the development of rehabilitation approaches targeting fear disorders.

## 5. Translational Perspectives

The relevance of neural oscillatory activity (e.g., delta, theta, gamma oscillations) that characterizes fear-related circuits is emphasized by the proposal of extending fear extinction exposure therapies so as to incorporate the artificial modulation (e.g., through man-made devices and training approaches) of theta/gamma activity related to prefrontal cortical–amygdalo–hippocampal–cerebellar pathways. To this end, the use of brain stimulation has recently been emphasized as a therapeutic approach that could potentially normalize abnormal oscillatory activity related to fear and anxiety disorders, such as PTSD and anxiety. In particular, recent human studies considered the use of electrical brain stimulation (tDCS and tACS) to the medial prefrontal cortex during extinction-learning. In contrast to the original hypothesis that anodal tDCS (“positive, usually enhancing”) would extinguish fear while low frequency tACS would reduce fear memory consolidation via a long-term depression mechanism, the authors reported that AC stimulation favored expression of the fear response, while DC stimulation led to overgeneralization of the fear response to non-reinforced stimuli. Thus, the need to perform additional studies to optimize parameters and stimulation sites to facilitate fear extinction for therapeutic purposes was emphasized [43]. In a different study, the authors reported an association between anodal tDCS (expected to have a positive effect on fear extinction) and accelerated late extinction learning of a second CS, after tDCS was combined with extinction learning of a previous CS. No significant effects of tDCS timing were observed on early extinction recall [44]. Interestingly, it has recently been reported that veterans suffering PTSD who received anodal tDCS over the vmPFC during extinction consolidation showed slightly lower skin conductance responses (SCR) in response to previously extinguished stimuli as compared to veterans who received tDCS simultaneous with extinction learning. No significant effect of tDCS on SCR during late extinction was reported [45].

Another neuromodulation approach that has been proposed in relation to fear extinction is vagal nerve stimulation (VNS). Recent studies in rats with induced PTSD have demonstrated that VNS has the potential to reverse extinction impairment and attenuate reinstatement (restoration) of the fear conditioning response. VNS also eliminated PTSD-like symptoms, such as anxiety, hyperarousal and social avoidance, from a week after VNS administration [46]. In healthy humans, a randomized-controlled trial showed that transcutaneous stimulation of the vagus nerve (tVNS) accelerated explicit fear extinction learning (as reflected by expectancy ratings of aversive stimuli occurrence that participants provided for each CS ) but did not lead to better retention of extinction memory 24 h later. Nevertheless, the authors were unable to assess the potential effects of tVNS on physiological indices of fear [47]. Other authors have reported generalized reinstatement in startle responses and differential reinstatement in valence ratings with no effect of tVNS on extinction or reinstatement [48].

Focusing on deep brain stimulation (DBS), it has been demonstrated that treatment of anxiety symptoms may be effective. For instance, obsessive compulsive disorder (OCD) patients treated with DBS have exhibited decreases in anxiety [49]. Interestingly, a recent case-report addressing neural oscillatory activity (STN-Local field potentials (LFP) and magnetoencephalography activity) from an OCD patient revealed local theta, alpha and beta oscillations in data recorded intraoperatively from both hemispheres, while data recorded postoperatively revealed high beta coherence between the STN and the sensorimotor cortex as well as theta-coupling between the STN and the anterior cingulate cortex [50]. Such theta coupling is in line with previous fear conditioning studies that have emphasized the role of theta oscillations in fear-related circuits. In fact, previous animal studies provided support for a beneficial effect of DBS in fear extinction, by targeting the dorsomedial ventral striatum (VS) while being administered during fear training [51]. As for the mechanism of such fear extinction, it was reported that DBS of the dorsal-VS increased Fos expression in the prelimbic and infralimbic prefrontal cortices and in the lateral division of the central nucleus of amygdala, an area that inhibits amygdala output [52].

Novel translational approaches for fear extinction also include decoded fMRI neurofeedback (pairing rewards with the occurrence of multi-voxel brain activity patterns matching a specific stimulus) combined with perceptual learning. In particular, a recent study showed the reduction of conditioned fear by pairing rewards with activation patterns in the visual cortex, representing a CS+. Interestingly, participants were unaware of the content and purpose of the feedback procedure so the use of unconscious processing was emphasized. As for a neural mechanism, successful extinction was related to disengagement of the vmPFC [53].

As exemplified by the mentioned translational approaches, the knowledge of oscillatory neural correlates of fear conditioning and extinction is key to fine tuning neurostimulation approaches targeting fear disorders. Nevertheless, one should keep in mind that proper optimization of a neuromodulation technique for the treatment of brain disorders relies not only on neural oscillatory markers but also several factors including neuropsychiatric, functional and anatomical factors, whose variability across subjects emphasizes the use of personalized medicine.
